# The usefulness of laparoscopic surgery for inguinal endometriosis

**DOI:** 10.1186/s40792-022-01571-x

**Published:** 2023-01-26

**Authors:** Shu Mushiake, Nao Kawaguchi, Mitsuhiro Asakuma, Koji Komeda, Tetsunosuke Shimizu, Fumitoshi Hirokawa, Tomoo Shimomura, Sang-Woong Lee

**Affiliations:** 1 Department of General and Gastroenterological Surgery, Osaka Medical and Pharmaceutical University Hospital, 2-7 Daigaku-Machi, Takatsuki, Osaka 569-8686 Japan; 2 Department of Surgery, Sousei Hospital, Kadoma, Osaka Japan

**Keywords:** Inguinal endometriosis, Laparoscopic surgery, TEP

## Abstract

**Background:**

Inguinal endometriosis is a rare clinical disease with an unclear etiology and pathogenesis, and its diagnosis requires accurate medical history-taking and histological examination. However, surgical treatment for the condition has not yet been standardized. This report presents two cases of inguinal endometriosis.

**Case presentation:**

The first patient was a 36-year-old woman who complained of pain and swelling in her right inguinal region. Physical examination revealed a soft, tender right inguinal mass. The size of the mass repeatedly increased and decreased during menstruation and did not show swelling with abdominal pressure. Magnetic resonance imaging showed a 3.5 × 2.5 cm mass with high intensity on T2-weighted imaging in the right inguinal canal, and no communication was found between the lesion site and the abdominal cavity. We diagnosed this case as inguinal endometriosis and managed it using an anterior approach and laparoscopic observation. The second patient was a 51-year-old woman who presented with an intermittently painful mass in her right inguinal region. The mass tended to increase in size, with worsening pain before menstruation. Abdominal computed tomography revealed a 2 × 2 cm cystic mass in the right inguinal region. We made a diagnosis of inguinal ectopic endometriosis and decided to operate via the totally extraperitoneal (TEP) method for excision plus transabdominal observation. The postoperative course in both cases was uneventful with no recurrence.

**Conclusions:**

Inguinal endometriosis is a rare entity that should be suspected in patients with cyclical symptoms of inguinal pain and swelling that correlate with their menstrual cycle, which might otherwise be attributed to inguinal hernia. It is crucial to make a preoperative diagnosis based on a careful medical review, physical examination, and imaging studies, and to make an appropriate surgical plan. Particularly, in the case of ectopic inguinal endometriosis involving the canal of Nuck, laparoscopic observation is useful for the intraoperative diagnosis of inguinal endometriosis to help rule out the involvement of other abdominal sites. However, it is important to select and modify the surgical technique to avoid rupturing the endometrisis mass and prevent postoperative recurrence.

## Background

Endometriosis is defined as the presence of normal endometrial mucosa outside the uterine cavity; its incidence in the inguinal region is reported to range from 0.07% to 0.8% [1, 2]. Clinicians may misdiagnose inguinal endometriosis as a hernia, lipoma, or hematoma. Preoperative diagnosis relies on a careful medical review and a detailed physical examination. Complete surgical excision is the curative treatment and prevents recurrence [3]. However, inguinal endometriosis is extremely rare, and its surgical procedure has not been standardized. Therefore, it is crucial to make a preoperative diagnosis and plan for proper surgery. We preoperatively diagnosed two cases of inguinal endometriosis and performed laparoscopic surgery. For each case, we considered the pathogenesis and proper surgical treatment, including the benefits of laparoscopy.

## Case presentation

### Case 1

**Fig. 1:**
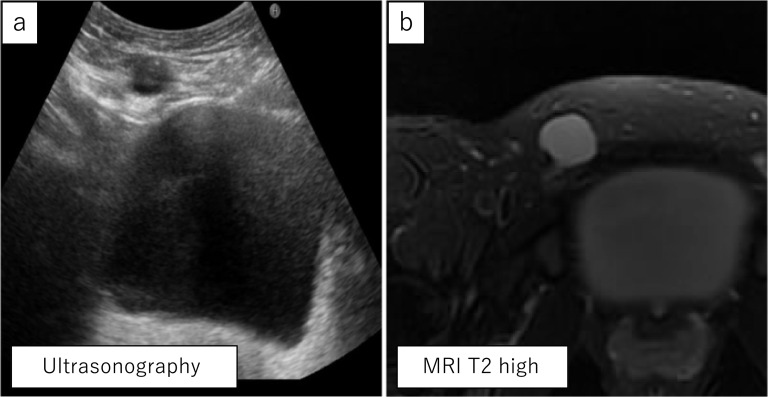
**a** Ultrasonography showed 3.0 × 2.0 cm cystic lesion. **b** Magnetic resonance imaging showed a 3.5 × 2.5 cm mass with high intensity on T2-weighted imaging in her right inguinal canal

A 36-year-old woman complained of pain and swelling in her right inguinal region, which appeared 5 months prior to presentation. Physical examination revealed a soft, tender right inguinal mass. A key historical feature was that the mass repeatedly increased and decreased with menstruation. The mass did not show swelling with maneuvers that increase abdominal pressure, such as coughing. Ultrasonography showed 3.0 × 2.0 cm cystic lesion and magnetic resonance imaging showed a 3.5 × 2.5 cm mass with high intensity on T2-weighted imaging in her right inguinal canal (Fig. 1), and no communication was found between the lesion site and the abdominal cavity. We diagnosed the case as inguinal endometriosis with no communication with the intra-abdominal cavity and decided to operate laparoscopically.

**Fig. 2:**
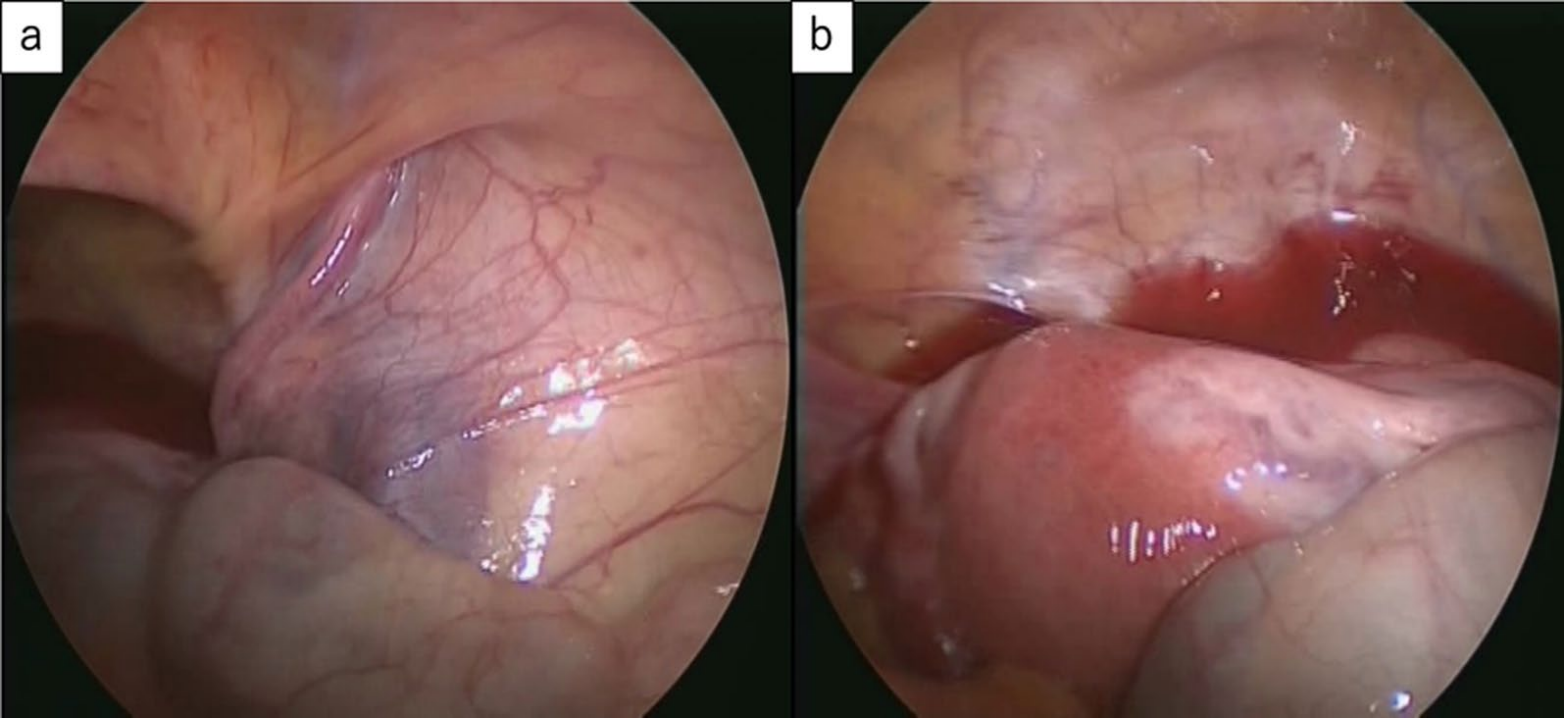
**a** Inguinal hernia that required treatment was not found on laparoscopic observation of the abdominal cavity. **b** Bloody ascites suggestive of endometriosis was found in the pouch of Douglas

**Fig. 3:**
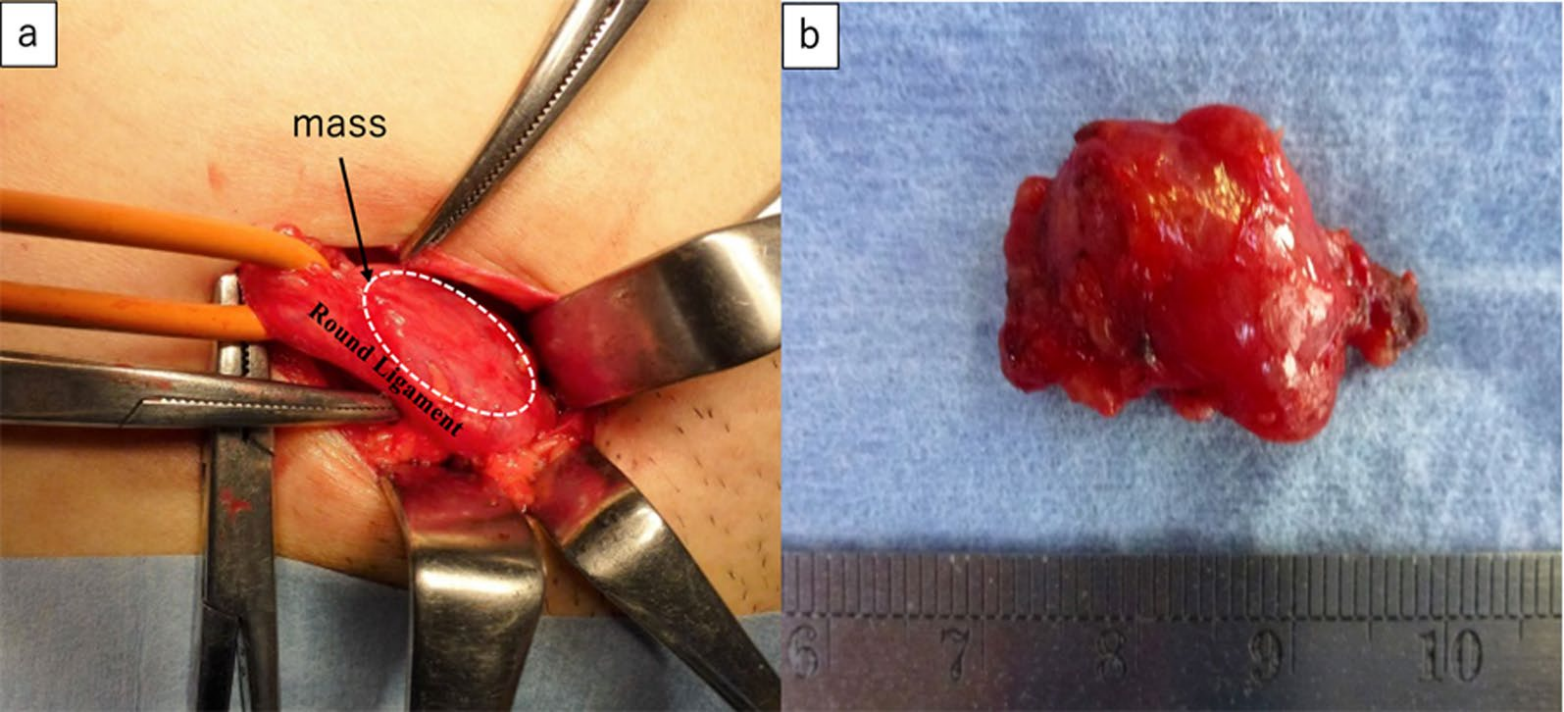
**a** Boundary between the mass and the surrounding tissue was well-defined. **b** Mass was removed without rupture

Intraoperative laparoscopy revealed the dilatation of the hernia gate in the right lateral abdominal wall similar to a slit. However, as the patient was a young woman, fertility preservation was prioritized, and we decided to not repair the hernia, but only to remove the mass (Fig. 2a). Bloody ascites suggestive of endometriosis was noted in the pouch of Douglas (Fig. 2b). We decided to remove the mass via an anterior approach rather than a laparoscopic approach, because the lesion was located near the pubis. The boundary between the mass and surrounding tissue structures was well-defined (Fig. 3a). We removed the mass without rupture (Fig. 3b) following laparoscopic observation and confirmed that the hernial gate was not dilated.

**Fig. 4:**
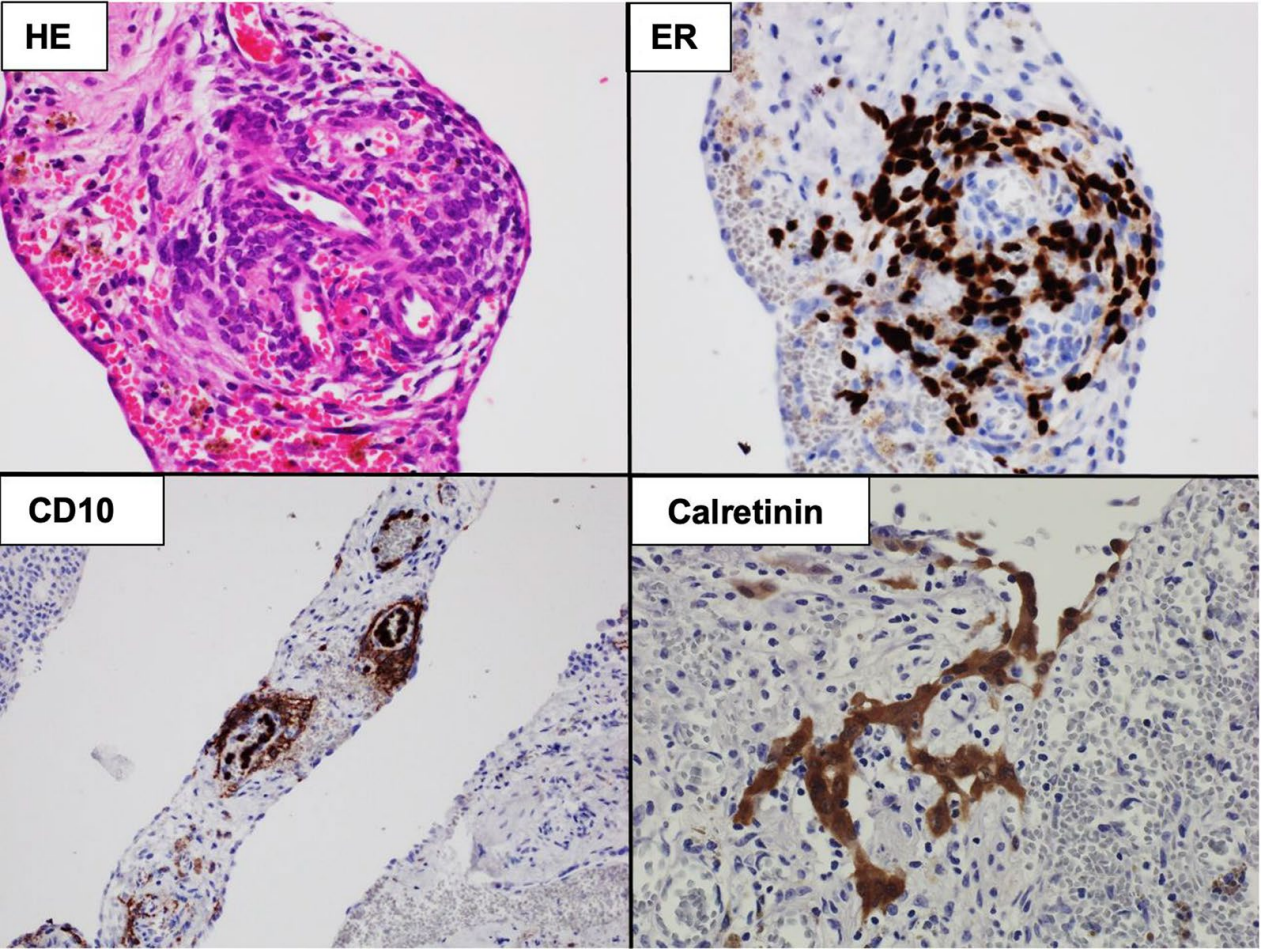
Collection of spindle-shaped cells in the subserosa on hematoxylin–eosin (HE) staining and positive estrogen receptor (ER) and CD10 immunostaining. Furthermore, the resected specimen was also positive for Calretinin stain

Histopathological examination revealed ectopic endometriosis in the canal of Nuck (Fig. 4). The patient was discharged without postoperative complications. Because bloody ascites was observed, and the presence of other sites of endometriosis was also suspected, the patient was closely followed up and has been without recurrence for 30 months postoperatively and the sympton of inguinal hernia has not been appeared.

### Case 2

**Fig. 5:**
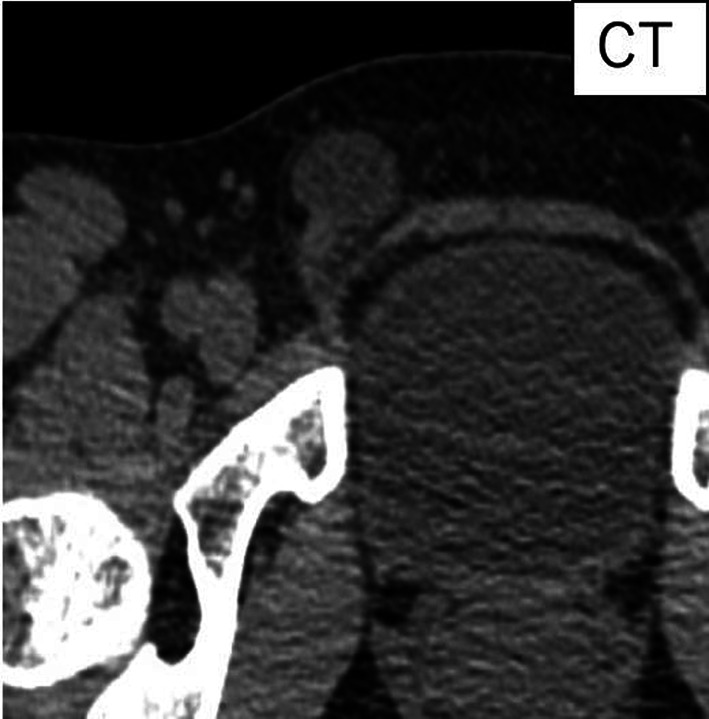
Abdominal computed tomography revealed a 20 × 20 mm cystic mass in the right inguinal region

**Fig. 6:**
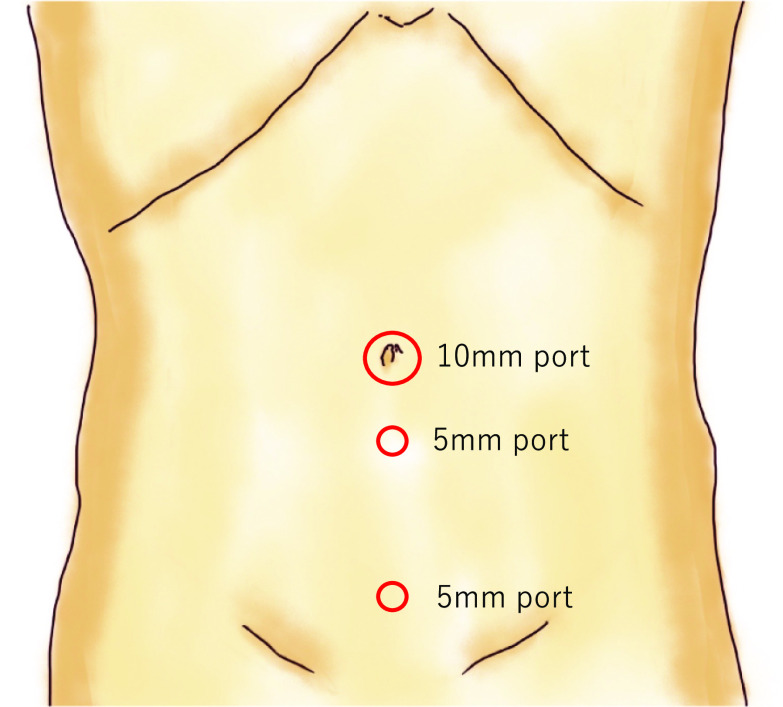
TEP ports placement after intraperitoneal observation. A 10 mm port at the umbilicus, 5 mm port above the pubis, and another midway between the pubis and the umbilicus

**Fig. 7:**
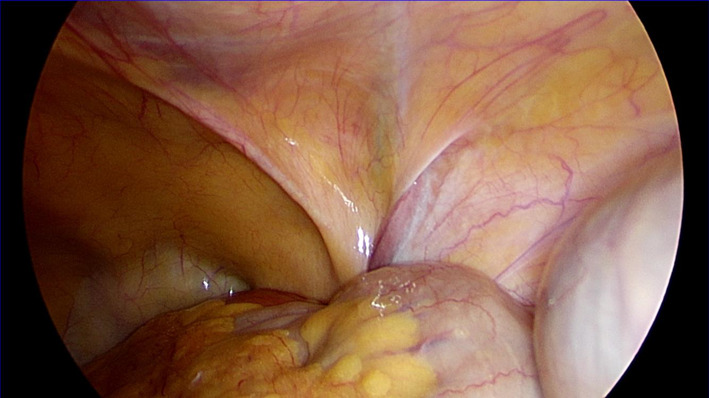
There was dilatation of the hernia gate (JHS classification; right I-1), and no ascites

**Fig. 8:**
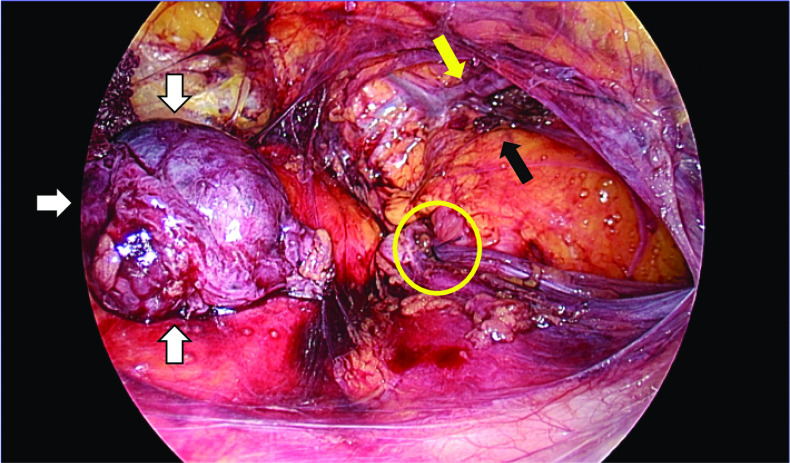
Excised specimen was a 20 × 20-mm cyst (white arrows), and the contents of the mass were bloody. The black arrow shows the hernia gate. Yellow circle is tip of hernia sac. Yellow allow is inferior abdominal artery and vein

**Fig. 9:**
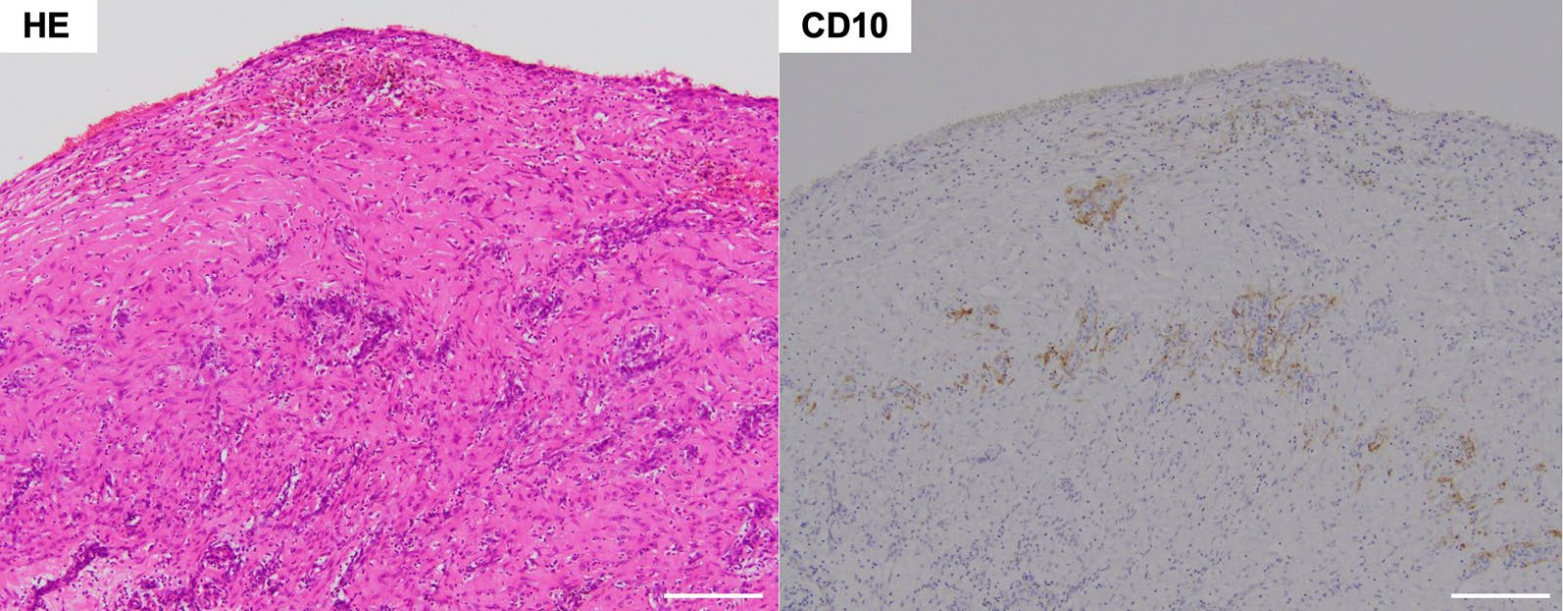
Hematoxylin–eosin (HE) staining showing congestion, hemorrhage, and infiltration of inflammatory cells and hemosiderin in the cell wall. The resected specimen was also positive for CD10 staining. Scale bar: 200 μm

A 51-year-old woman presented with an intermittently painful mass in her right inguinal region. The mass tended to increase in size, and the pain worsened before menstruation. Abdominal computed tomography revealed a 2 × 2 cm cystic mass in her right inguinal region (Fig. 5). We made a diagnosis of inguinal ectopic endometriosis. We decided to excise the endometriotic lesion via the totally extraperitoneal (TEP) method in addition to transabdominal observation. We inserted three trocars; a 10 mm trocar at the umbilicus, a 5 mm above the pubis, and another midway between the pubis and the umbilicus (Fig. 6). Laparoscopy revealed the absence of bloody ascites and the presence of right lateral abdominal wall dilatation of the hernia gate (JHS classification; right I-1) (Fig. 7). We isolated the mass after confirming that only the round ligament remained on the periphery (Fig. 8). After excision, we spread the mesh to cover the hernia gate and reviewed the abdominal cavity again. Histopathological examination revealed endometriosis (Fig. 9). The patient tolerated the procedure well with no complications, and has had no recurrence after 20 months of follow-up.

## Discussion

Inguinal endometriosis is a rare condition, and its etiology and pathogenesis remain unclear. Several etiological theories exist regarding the developmental mechanism of endometriosis in the inguinal region. The first is the “transplantation theory” of lymphatic or hematogenous spread. The second theory is the “direct extension theory” of direct invasion to the inguinal region. The third is the “metaplasia theory,” in which endometrial tissue from the Müllerian ducts causes metaplasia of the peritoneal epithelium [2, 4]. In this report, both cases had a well-defined boundary between the round ligament and the mass, and the first case also involved the canal of Nuck. These facts may be a reasonable basis for attributing their inguinal endometriosis to the metaplasia theory. It is interesting that Wolfhagen et al. also demonstrated the canal of the Nuck in their resection specimens, which is possibly an essential key to pathogenesis [5].

In the preoperative diagnosis of inguinal ectopic endometriosis, it is important to interview the patient to determine whether inguinal pain becomes more severe with menstruation. Although we did not measure CA125 (a blood marker for endometriosis) in our study, CA125 was elevated in approximately half of the cases [6]. It may appear that elevated blood CA125 is useful for preoperative diagnosis. As for the treatment plan, ectopic endometriosis is generally treated with surgery, symptomatic treatment, endocrine therapy, or approaches that combine these modalities [4]. To our knowledge, there is no established surgical technique for inguinal ectopic endometriosis, but it is important to prevent the spread of the lesion to surrounding organs when removing the mass.

In this report, the first case was managed using the anterior approach plus laparoscopic observation, and the second case was managed using the TEP method plus abdominal observation, which combines the merits of both the transabdominal peritoneal approach (TAPP) and TEP. An advantage of laparoscopic surgery is that it can be used to detect pelvic endometriosis, which can lead to the recurrence of inguinal ectopic endometriosis [5]. Laparoscopy can also be used to directly investigate for the presence of an inguinal hernia, which may be associated with inguinal endometriosis [7, 8]. The treatment of endometriosis requires complete resection of the endometriotic lesion. In the case of ectopic endometriosis, complete excision of the round ligament by laparoscopic surgery may be useful if the lesion extends into the abdominal cavity via the round ligament [9]. In the case of inguinal ectopic endometriosis involving the canal of the Nuck, it is important to completely remove the mass. Below, we consider whether the TAPP or TEP method is more suitable for laparoscopic surgery. In the TAPP method, it is possible that the peritoneal sheath is pulled into the inguinal canal because of pneumoperitoneum. Dissecting the peritoneal sheath on the peripheral side of the inguinal canal may be difficult. In contrast, in the TEP method, the approach is performed through the extraperitoneal space without incising the peritoneum, which allows sufficient dissection to the peripheral side of the inguinal canal without interfering with the abdominal wall structure in the groin. Regarding dissection to the peripheral side of the inguinal canal, if the mass ruptures intraoperatively, the endometriosis lesion will spread. The TEP method may reduce the risk of intraoperative damage to the mass due to its magnifying effect. In addition, the TEP method may reduce the effect of endometriosis lesions on the abdominal cavity considering it does not incise the peritoneum. Hence, the TEP method may be superior to the TAPP method in terms of postoperative safety and a low risk of recurrence.

It is important not to rupture the mass, but to remove its contents without seeding the surrounding tissue [10]. If the size of the lesion is larger than the umbilical incision and it is difficult to remove the mass without rupturing it, the operative approach should be modified by adding an anterior approach as shown in Case 1.

Finally, laparoscopic observation of the abdominal cavity is useful for the preoperative diagnosis of inguinal endometriosis to help rule out the involvement of other abdominal sites, as the disease has the potential to recur or undergo malignant transformation [8, 11].

## Conclusions

Inguinal endometriosis is a rare entity that should be suspected in patients with cyclical symptoms of inguinal pain and swelling that correlate with their menstrual cycle, which might otherwise be attributed to an inguinal hernia. It is crucial to make a preoperative diagnosis from a careful medical review, physical examination, and imaging studies, and to make an appropriate surgical plan. When performing surgery for inguinal endometriosis, laparoscopic observation of the abdominal cavity is useful for the preoperative diagnosis to help rule out the involvement of other abdominal sites, considering the possibility of recurrence or malignant transformation. It is important to select and modify the surgical technique to avoid rupturing the mass and prevent postoperative recurrence.

## Data Availability

Not applicable.

## Abbreviations

TAPP: Transabdominal peritoneal approach
TEP: Totally extraperitoneal
